# Wild bonobos experience unusually low bone resorption during early lactation relative to humans and other mammals

**DOI:** 10.1017/ehs.2025.10013

**Published:** 2025-07-22

**Authors:** Verena Behringer, Ruth Sonnweber, Barbara Fruth, Genevieve Housman, Pamela Heidi Douglas, Jeroen M. G. Stevens, Gottfried Hohmann, Tracy L. Kivell

**Affiliations:** 1 Endocrinology Laboratory, German Primate Center, Leibniz Institute for Primate Research, Göttingen, Germany; 2 Department of Behavioral and Cognitive Biology, Faculty of Life Sciences, University of Vienna, University Biology Building (UBB), Vienna, Austria; 3 Max Planck Institute of Animal Behavior, Konstanz, Germany; 4 Centre for Research and Conservation, Royal Zoological Society of Antwerp, Antwerp, Belgium; 5 Department of Primate Behavior and Evolution, Max Planck Institute for Evolutionary Anthropology, Leipzig, Germany; 6 Language Centre, Paderborn University, Paderborn, Germany; 7 SALTO Agro- and Biotechnology, Odisee University of Applied Sciences, Sint-Niklaas, Belgium; 8 Department of Human Origins, Max Planck Institute for Evolutionary Anthropology, Leipzig, Germany

**Keywords:** Reproductive phase, hominoid, CTX-I, bone turnover markers, *Pan paniscus*, bone density

## Abstract

In mammals, pregnancy and lactation are marked by maternal calcium stress and bone resorption, leading to reduced bone mineral density. In humans, these periods may partly explain the higher prevalence of osteoporosis in older women compared with men, but lactation patterns in modern humans may reflect cultural influences rather than natural conditions. The extent to which these findings apply to wild-living mammals remains unknown. We measured urinary C-terminal crosslinking telopeptide of Type I collagen (CTX-I) levels, a bone resorption marker, during pregnancy in wild and zoo-housed bonobos (*Pan paniscus*) and during lactation in wild bonobos. Studying wild-living primates such as bonobos can provide insights into ancestral reproductive adaptations. We found an increase in CTX-I levels towards the end of pregnancy in zoo-housed and primiparous wild females. Contrary to expectations, CTX-I levels during early lactation are lower than in other reproductive phases. This pattern diverges from the assumption that lactation increases bone resorption. Our findings suggest that wild bonobos may rely on a combination of physiological and behavioral strategies to modulate bone metabolism during lactation. Bone resorption may serve as a physiological back-up when behavioral or dietary strategies cannot fully meet calcium demands. These flexible responses, shaped by fluctuating environmental conditions and prolonged maternal investment, provide insight into evolutionary pressures on skeletal health and may inform strategies to mitigate bone loss in humans.

## Graphical abstract

**Figure:**
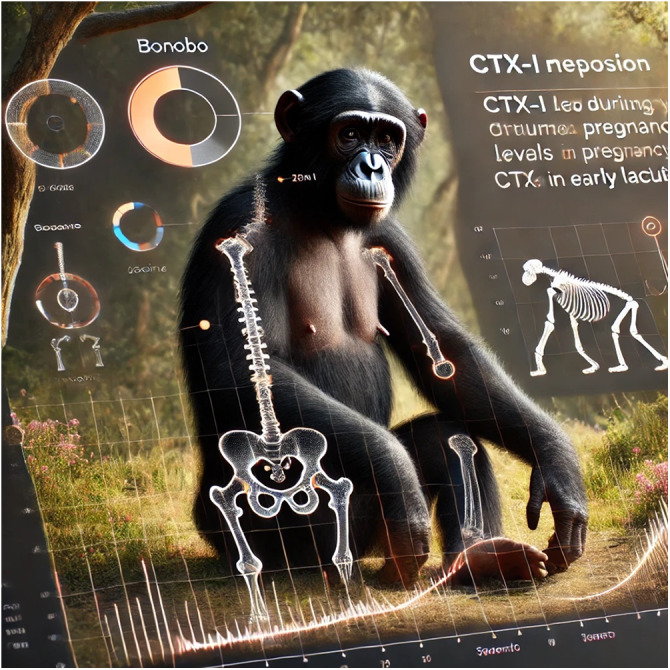

## Social media summary

In wild bonobos, during early lactation bone resorption is unexpected low, revealing unique adaptations.

## 1. Introduction

In humans, bone loss and osteoporosis, characterized by reduced bone density and increased fracture risk, profoundly impact health and impose substantial burdens on healthcare systems (Akkawi & Zmerly, 2018; Salari, 2014). These conditions disproportionately affect older women from well-studied populations of the global north (Sözen et al., 2017). In women, reduced bone mineral density (BMD) has been associated with aspects of reproduction, including short interbirth intervals and prolonged lactation periods (Akkawi & Zmerly, 2018; Salari, 2014; Stieglitz et al., 2015). These stages involve complex physiological changes, yet much of what we know about bone metabolism is based on studies of modern humans, as well as some domesticated and wild mammal species living in captivity. Over the course of human evolution, reproductive strategies such as lactation patterns have changed (Sellen, 2007), potentially influencing bone metabolism in ways that are not yet fully understood. Likewise, non-human mammals living under controlled conditions may evolve metabolic traits that differ from wild conspecifics. Studying bone metabolism during these life stages in wild non-human primates provides valuable insights into evolutionary processes that may have shaped these mechanisms in humans.

In mammals, changes in calcium metabolism occur during pregnancy and lactation to meet foetal and offspring skeletal growth demands (Bowman & Miller, 2001; Kovacs, 2011; Szulc et al., 2017). In humans, calcium transfer to the foetus involves increased intestinal absorption, modulation of renal calcium losses, and, to a lesser extent, resorption from maternal bones (Kaur et al., 2003; Sowers, 1996). Lactation further intensifies these demands as calcium is secreted into milk (Wysolmerski, 2003), inducing a temporary demineralization of the skeleton as the primary mechanism to fulfil calcium requirements (Kovacs, 2011; Sowers et al., 1996). A remarkable 10% reduction in bone mass has been observed in women who exclusively breastfeed (Kovacs, 2011). However, studies have shown that this bone loss is largely reversible. Most women experience a full or near-full recovery of BMD after weaning and that lactation does not have long-term adverse effects on peak bone mass, bone density, or fracture risk (Augustine et al., 2023; Kovacs, 2001; Kovacs & Kronenberg, 1997; Madimenos et al., 2012; but see Affinito et al., 1996; Brembeck et al., 2015). These results suggest that lactation-induced bone depletion in humans is a transient physiological process rather than a cumulative deficit.

Similar calcium demands and bone loss patterns during the postpartum period have been shown in captive non-human primates (e.g. Lees et al., 1998; Ott et al., 1999) and domesticated mammals (e.g. Liesegang et al., 2007; Van Riet et al., 2016). However, calcium demands bone resorption dynamics during pregnancy and lactation have yet to be studied in any wild-living mammal, leaving a critical gap in our understanding of the physiological costs of reproduction for females in natural living conditions. We address this gap through a non-invasive investigation of the physiological impact of pregnancy and lactation on bone resorption in wild-living bonobos (*Pan paniscus*), using a biomarker of bone turnover sampled from urine.

### 1.1. Bone turnover biomarkers

Urinary bone resorption biomarkers, such as C-terminal crosslinking telopeptide of Type I collagen (CTX-I), offer a non-invasive method to quantify bone turnover within the living skeleton and to assess how pregnancy and lactation, in particular, impact bone turnover (Liesegang et al., 2007). Biomarkers such as CTX and NTX (N-terminal crosslinking telopeptide) are increasingly used for assessing bone turnover, as they are released into circulation in proportion to collagen degradation by osteoclasts (Herrmann & Seibel, 2008; de Ridder & Delemarre-van de Waal, 1998; van der Sluis et al., 2002). This is largely because over 90% of the organic matrix of bone consists of type I collagen (Vasikaran, 2008; Yamaga et al., 1997), and collagen crosslinks such as NTX-I and CTX-I are direct degradation products of this collagen (Herrmann & Seibel, 2008; Rosen et al., 2000). CTX is a small peptide fragment cleaved from type I collagen and thus can be measured in urine (Herrmann & Seibel, 2008; Urlacher et al., 2022). Therefore, the urinary excretion rate of crosslink-containing collagen fragments serves as an indicator of bone collagen degradation (Mora et al., 1998), offering a non-invasive method to evaluate bone resorption.

To our knowledge, only one other study has assessed bone turnover in a wild primate: Sandel et al. (2023) analysed urinary biomarkers, including osteocalcin (bone formation marker) and NTX (bone resorption marker), in a cross-sectional sample of wild chimpanzees (Ngogo, Kibale National Park, Uganda) to investigate skeletal growth during ontogeny. The study found that bone turnover biomarkers displayed a nonlinear relationship with age, with males showing distinct peaks in osteocalcin and NTX levels during adolescence (at 9.4 and 10.8 years, respectively), followed by a plateau at around 20 years in both sexes. Sandel et al. (2023) interpreted these findings as indicative of a potential adolescent growth spurt in chimpanzees, particularly in males, challenging the long-held assumption that such growth spurts are unique to humans. However, the study did not assess changes in bone turnover in females related to pregnancy or lactation.

### 1.2. Bone turnover during pregnancy and lactation in humans

During human pregnancy, maternal urinary CTX-I levels increase two- to threefold, reflecting increased bone resorption as the foetal skeleton develops (reviewed in Hannon & Eastell, 2000). Similarly, bone turnover markers fluctuate during human lactation. This includes changes in bone formation markers such as amino-terminal telopeptides of procollagen type I and osteocalcin, as well as the bone resorption markers NTX and CTX-I (Carneiro et al., 2010; Møller et al., 2013; Prentice et al., 1998). Postpartum levels of these markers increase several-fold and remain elevated during lactation, often even exceeding levels observed in late pregnancy (Kovacs, 2011; Yamaga et al., 1997).

Although studies have explored how pregnancy and lactation affect bone turnover markers in healthy women, most research has focused on populations in the global north (Augustine et al., 2023; Carneiro et al., 2010), which may not be universally representative. Research quantifying BMD in non-global north populations is more common. For example, BMD studies of the Shuar women of Amazonian Ecuador (Madimenos et al., 2012) and cross-population comparisons of the Tsimane and Shuar (Madimenos et al., 2020) have provided valuable insights into the reproductive effects – and the variation of those effects – on skeletal health. Other studies have revealed differences in both baseline BMD and how BMD changes during pregnancy and lactation. For instance, compared with self-identified Caucasian mothers, African American mothers exhibit differences in baseline BMD and less lactation-induced bone loss at certain skeletal sites, such as the lumbar vertebrae and femoral neck (Augustine et al., 2023).

### 1.3. Bone turnover during pregnancy and lactation in non-human mammals

**Figure 1.**
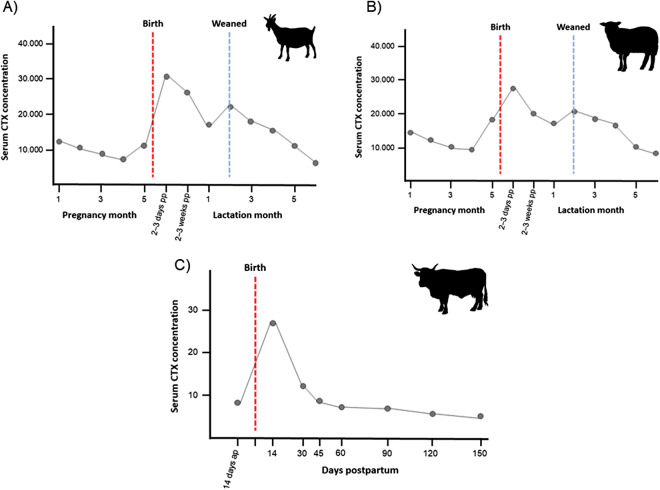
Schematic diagrams of the bone resorption marker CTX during pregnancy and lactation in domesticated (a) goats, (b) sheep, and (c) cows. Goat and sheep plots modified after Liesegang et al. (2006), showing the timing of birth and weaning of offspring. Cow plot modified after Liesegang et al. (2000), showing just the timing of birth, as data on weaning were not provided in the study. ap = ante partum; pp = postpartum.

Differences in bone turnover patterns during pregnancy have also been documented across a variety of species. Similar to humans, domesticated animals exhibit increased serum CTX-I levels before parturition (Liesegang et al., 2000, 2006, 2007; Van Riet et al., 2016; Figure 1). However, studies of captive monkeys show varied patterns during pregnancy: southern pig-tailed macaques (*Macaca nemestrina*) exhibit significant changes in bone metabolism (Ott et al., 1999), whereasile long-tailed macaques (*Macaca fascicularis*) show no change in biomarker levels (Lees et al., 1998). Although biomarkers have not yet been quantified, a study measuring BMD directly in female rhesus macaques (*Macaca mulatta*) from Cayo Santiago showed that BMD increased with the number of offspring produced (Cerroni et al., 2003). Together, these findings illustrate the diversity of bone turnover responses during pregnancy and emphasize the importance of species-specific and ecological factors in shaping these patterns.

Bone formation markers have also been investigated in various non-human mammals, revealing patterns similar to those found in humans. These patterns have been observed in sows (Van Riet et al., 2016), goats and sheep (Liesegang et al., 2006), mice (VanHouten & Wysolmerski, 2003), little brown bats (*Myotis lucifugus*; Kwiecinski et al., 1987), and dogs (M. A. Miller et al., 1989) (Figure 1). Captive monkeys (*M. fascicularis, M. nemestrina, Chlorocebus aethiops, Callithrix jacchus*) also show increased bone resorption and remodelling during lactation, with partial bone calcium depletion (Hiyaoka et al., 1996; Lees et al., 1998; Ott et al., 1999; Power et al., 1999). These interspecies comparisons highlight the conserved physiological processes of bone metabolism during lactation across different mammalian species, but corresponding data for apes are currently lacking.

Despite the notable research, it is unclear to what extent these findings can be extrapolated to other modern human populations or to other, primarily wild-living, mammals. For example, in other non-global north human populations, including hunter-gatherers and pastoralists (Mays, 2016), bone metabolism may be influenced by lifestyle factors, including nutrition, offspring feeding strategies, and reproductive histories (Mays, 2016; Sellen, 2007).

Similarly, in other mammals, data are derived mainly from domesticated and captive animals; however, the activity levels, disease and contraception management practices, as well as the behaviours of these animals differ from their wild-living counterparts (Beaulieu, 2016; Calisi & Bentley, 2009). Studies comparing captive and wild individuals have shown the profound impacts of the environment on physiological outcomes (Beaulieu, 2016; Turko et al., 2023). For example, captive conditions, such as temperature, light, and humidity, may introduce artificial stressors that can potentially skew the assessment of physiological parameters (Beaulieu, 2016). Factors such as parasite load, stressor, diet quality, quantity, and feeding schedule can all differ between captive and wild settings, further altering physiological outcomes (Turko et al., 2023). Among primates, wild-living baboons have lower thyroid hormone levels during pregnancy compared to the cycling phase, a pattern that contrasts with findings in captive primates (Gesquiere et al., 2018). The authors suggested that elevated thyroid hormone levels commonly observed during pregnancy in well-nourished women may not be applicable to undernourished populations (Gesquiere et al., 2018), highlighting the significant role of ecological factors in shaping physiological regulation. Moreover, activity patterns, muscle mass, or body weight can influence bone structure, resulting in differences between species or captive versus wild individuals of the same species in the baseline amount of bone structure (e.g. differences in cortical bone thickness or volume of trabecular bone) (Chirchir et al., 2022; Harbers et al., 2020; Pelletier et al., 2021; Zack et al., 2022).

### 1.4. Maternal investment and bone resorption dynamics across species

Across species, maternal investment is highly variable, influencing calcium allocation patterns and potentially affecting bone resorption. In species with precocial young (e.g. cows, sheep), offspring are mobile shortly after birth and require less prolonged care. In contrast, primates have altricial young and exhibit extended maternal care, particularly through prolonged lactation, which maintains calcium demands over an extended period and may lead to sustained bone resorption. With their slow life history, primates, especially apes and humans, experience extended infant dependency, with maternal care and nutritional support spanning multiple seasons (Charnov & Berrigan, 1993; van Noordwijk et al., 2013). However, humans differ from great apes in several aspects: human infants are born heavier, with more body fat; human milk is frequently supplemented with additional foods; infants receive their first solid foods prepared by caregivers; and the lactation period is shorter in most human populations, leading to an earlier weaning age compared with great apes (van Noordwijk et al., 2013). In primates, the prolonged maternal investment exposes primates to fluctuating resource availability (van Noordwijk et al., 2013), requiring mothers to balance their own reserves, including bone resorption, to meet offspring bone growth demands. This dynamic balancing of bone turnover is particularly relevant in species with long lactation periods, where environmental conditions and maternal energy balance may influence bone metabolism.

Bonobos exhibit an exceptionally prolonged period of maternal investment, characterized by long gestation, extended lactation, and extensive infant care. Pregnancy lasts approximately 240 days, similar to humans, but lactation is prolonged, typically lasting 4–5 years (Douglas, 2014; Jukic et al., 2013; Oelze et al., 2024; Stevens, 2020). During this time, offspring continue to rely on their mothers for both nutritional and social support, with milk remaining an important source even as solid food intake gradually increases (Oelze et al., 2024). Additionally, bonobo infants are carried by their mothers for more than a year (Behringer et al., 2022; Lee, Hohmann, et al., 2020; Lee, Murray, et al., 2020). This maternal investment strategy differs markedly from that of domesticated species frequently studied in bone resorption research, such as pigs, goats, and sheep (Liesegang et al., 2006; Van Riet et al., 2016). In these species, weaning occurs within months (often earlier under artificial conditions), and offspring rapidly gain independence, reducing the duration of direct maternal care (Alley et al., 1995; Freitas-de-Melo et al., 2022). Compared to human populations in which bone resorption markers have been measured (see above), bonobos exhibit even longer lactation durations. This extended lactation in wild bonobos likely involves prolonged maternal calcium allocation, potentially resulting in a distinct bone resorption pattern compared to humans and domesticated mammals.

Here, we conducted the first investigation into maternal physiological costs during pregnancy and lactation on bone resorption in a wild-living animal, the bonobo. Investigating bone metabolism during pregnancy and lactation in bonobos can enhance our understanding of the evolutionary adaptations involved in maternal skeletal responses to reproduction. This can contribute to comparative insights into the mechanisms of bone turnover and calcium regulation in primates, with implications for human evolutionary biology. For biological validation, we first measured urinary CTX-I in two pregnant bonobos in a controlled zoo environment with a high and regular sampling frequency (*n* = 86 samples) and compared these data with published results from other captive animals and humans. Biological validation, in the context of our study, refers to demonstrating that urinary CTX-I measured in bonobos shows a pattern that corresponds to expected physiological changes, such as those observed during pregnancy and lactation in other mammals. This verifies that the assay detects biologically meaningful variation in urinary CTX-I levels, consistent with established knowledge of bone metabolism in other species, and ensures that these changes align with key life-history stages in bonobos. We then quantified changes in urinary CTX-I levels in pre-pregnant, pregnant, and lactating wild-living bonobos (*n* = 10 individuals, *n* = 384 samples, Table S1).

If the human and domesticated species pattern is typical for all mammals under diverse conditions, we predicted that urinary CTX-I levels, a marker of bone resorption, will progressively increase during pregnancy and peak in late gestation. Such a pattern would indicate increased bone turnover, consistent with findings in modern humans as well as captive and domesticated mammals. However, if wild mammals have evolved alternative strategies to meet calcium demands during pregnancy and lactation, urinary CTX-I levels might remain stable rather than increase. Additionally, we predicted higher urinary CTX-I levels in lactating compared to pregnant or cycling females, assuming lactation involves increased maternal bone resorption to meet calcium demands for milk production, as observed in other species. However, cultural factors in humans, such as diet and physical activity, may alter this pattern. If the timing, intensity, and duration of maternal investment influence bone resorption, as suggested in previous studies (see above), then we expected bone resorption to fluctuate throughout pregnancy and lactation in wild bonobos as mothers adjust to variation in resource availability throughout this extended period of investment.

Information on natural calcium resources in tropical forests is scarce; however, it is generally assumed that calcium content is low due to leaching (Gonçalves Bizuti et al., 2018). If this assumption applies for the conditions at LuiKotale, home to our wild study population, it can be argued that calcium content in the LuiKotale forest is limited, potentially constraining the capacity for bone remodelling and thereby necessitating physiological adjustments in females. During late pregnancy, when calcium demands are high, we might see an increase in urinary CTX-I levels. However, in contrast to domesticated or controlled environments, wild bonobos may exhibit a more moderate increase in bone resorption, reflecting adaptive strategies such as increased calcium absorption or retention. During lactation, CTX-I levels would likely be higher, as calcium demands for milk production peak. However, these levels might vary throughout lactation, reflecting the dynamic balancing act between conserving bone mass and meeting the energy and calcium demands of offspring bone growth. This balance would likely depend on the environmental conditions and the maternal energy balance.

## 2. Methods

### 2.1. Study species and subjects

Urine samples for this study originate from two distinct sources. The first data set comprises samples from two zoo female bonobos housed at Planckendael Zoo (Mechelen, Belgium), which were collected exclusively during their pregnancies as part of an earlier study (Heistermann et al., 1996). These samples were included in the present study for biological validation purposes, specifically to demonstrate that if a pregnancy-associated pattern in urinary CTX-I levels exists, it can be reliably detected in bonobos. Although lactation samples are unavailable for these individuals, the inclusion of these pregnancy samples is crucial for confirming detectable changes in CTX-I during pregnancy.

The second data set consists of samples collected from 10 wild female bonobos living in their natural habitat at LuiKotale, Democratic Republic of the Congo (DRC). These samples represent all three reproductive states – pre-pregnancy, pregnancy, and lactation – and were collected over a 10-year period.

#### Zoo-housed bonobos (biological validation)

Two multiparous female bonobos from Planckendael Zoo were housed in a single group, fed several times daily, and given *ad libitum* access to water. In total, 86 urine samples were collected from these individuals. For one female, 35 samples were collected in 1994. For the other female, 51 samples were collected between May 1992 and the birth of the offspring in February 1993. Both pregnancies were successful. Because these samples were collected as part of a validation study on the cycle and pregnancy of bonobos (Heistermann et al., 1996), there are no samples from the lactation period. However, due to the high sample density during pregnancy, we included these samples in the study to evaluate CTX-I levels under controlled conditions. Samples were collected on plastic sheets or from the floor with disposable plastic pipettes and transferred into 2 ml plastic vials. Urine was frozen immediately after collection. All samples were collected in the morning, at approximately the same time for each female.

Long-term stability studies on urinary CTX-I are lacking; however, bone resorption markers, including CTX-I, are generally stable when frozen (Szulc et al., 2017). Furthermore, plasma and serum CTX-I have been shown to remain unaffected by storage at −20°C for up to three years (Qvist et al., 2004). Although some of our samples were stored for over 30 years before analysis, CTX-I was consistently detected in our samples, and the observed patterns align with physiological expectations. This suggests that if degradation has occurred, it has not precluded reliable measurement.

#### Wild-living bonobos

Urine samples were collected from wild-living bonobos of the Bompusa West and East communities at LuiKotale, DRC. This bonobo population has never been provisioned with food and lives in an intact, natural forest habitat (Hohmann & Fruth, 2003). All subjects were individually known and habituated to human presence. Samples were collected opportunistically throughout the day, between 05:00 and 18:00, by capturing urine directly or pipetting urine from the vegetation, and were stored frozen.

Following the birth of an infant, we are confident that the urine samples collected belong to the mothers, rather than the infant, for the following reasons. First, urine from infant bonobos typically has a very low specific gravity, and samples with such low values were excluded from our analysis (<1.003; Sabbi et al., 2020). Second, in the context of another ongoing study, we measured sex steroids in the samples, which indicated that they came from reproductively active females.

A total of 384 urine samples from 10 female bonobos (on average 38 ± 13.7 samples per individual, range 22–68) were collected between 2011 and 2022. Samples were collected while females were cycling, pregnant, and lactating. All pregnancies included in our data set had a successful outcome. Nine of the 10 females included in the data set had one pregnancy; one female had two pregnancies. Therefore, in total, our data set encompassed 11 pre-pregnancy, pregnancy, and lactation periods from 10 females. Importantly, the sampling period for each female was limited to one or (in one case) two reproductive cycles and did not extend across the entire 10-year study period (for details regarding sample numbers, see Table S1). Five females were primiparous and five were multiparous. The females’ exact ages are unknown, because females are the dispersing sex in bonobos (Eriksson et al., 2006; Ishizuka et al., 2020). However, all primiparous females are estimated to be younger than 20 years of age and all multiparous females are older than 25 years.

Birthdates of all offspring were estimated based on three criteria: (1) the date of the female’s last observed encounter without offspring, (2) the date of the first observed encounter with new offspring, and (3) the developmental stage of the new born at first sighting. Estimates range from a few days up to a few weeks. We used the birthdate of each offspring as a reference point for timelines within females. All dates when a urine sample was collected from a given female were assigned a negative value (before pregnancy or during pregnancy) or a positive value (lactation) relative to the offspring’s birthdate (the number of days from the birthdate to the sample date). Samples were collected up to 765 days before and up to 667 days after a birth. As the average gestation length in bonobos is approximately 245 days (Stevens, 2020), we assumed −245 days from the birthdate to be the beginning of a pregnancy. All samples collected after the birthdate of an offspring (up to 667 days after birth) are from lactating females. However, the majority of urine samples during the lactation period were collected within the first 6 months after an offspring’s birth (for details, see Figure S1).

Our first data set included CTX-I levels during pregnancy and lactation. This data set encompassed 296 urine samples from 10 females. To achieve a more symmetrical distribution of our response variable, we log-transformed urinary CTX-I levels. The time of sample collection and the days relative to birth were mean-centred and standardized to two standard deviations.

Because we found an unexpected result at the beginning of the lactation period, we performed further analyses. To that end, we included the data collected pre-pregnancy to create a second data set (details in Table S1). Samples collected between −765 and −246 days relative to offspring birth were assigned to the pre-pregnancy period, samples collected between −245 and 0 days relative to offspring birth were assigned to the pregnancy period, and values greater than 0 were assigned to the lactation period. We calculated pregnancy trimesters by dividing the gestation length into thirds: samples collected on days 1–81 were assigned to the first trimester, those on days 82–162 to the second trimester, and samples on days 162–245 to the third trimester. Furthermore, we divided the lactation period into early lactation (from birth to 90 days after birth) and late lactation (more than 90 days after birth).

### 2.2. CTX-I measurement

Urine samples from LuiKotale were shipped to the Max Planck Institute for Evolutionary Anthropology (MPI EVA) in Leipzig, Germany. The samples were shipped frozen to the German Primate Center in Göttingen, Germany, for CTX-I measurement.

For CTX-I measurement, we used the commercial sandwich assay (two highly specific antibodies) Urine BETA CrossLaps® (CTX-I) ELISA (REF AC-05F1) from Immunodiagnostic Systems (IDS). The assay is designed for quantifying degradation products of C-terminal telopeptides of Type I collagen in human urine and was previously validated for use in bonobo urine (Behringer et al., 2024). The assay description is in Supplemental Material S1. The results are expressed in ng/ml corrected for specific gravity. In all samples, specific gravity was measured using a digital hand refractometer to adjust for urine concentration, which is based on individual hydration status (Miller et al., 2004).

### 2.3. Statistical analyses

We expected urinary CTX-I levels to vary in a non-linear fashion within and across different reproductive phases in female bonobos. Consequently, we ran generalized additive mixed effect models (GAMMs) as these allow for tracking complex, non-linear relationships (smooths) between the response variable and different predictors (Table S2). All GAMMs were specified with a Gaussian distribution and an identity link using the ‘gam’ function of the package ‘mgcv’ (Wood, 2017). Smooth estimation was based on maximum likelihood estimation, and the smooth basis was set to penalized cubic regression splines. These and all other data preparations and statistical analyses were performed using R v. 4.2.3 (R Development Core Team, 2023) in RStudio v. 2023.06.1+524 (R Studio Team, 2020). The threshold for statistical significance was set at *p* = 0.05.

Our main model included log-transformed urinary CTX-I levels as the response variable. As CTX-I levels follow a circadian pattern (reviewed in Hannon & Eastell, 2000), we included the time of sample collection (mean-centred and standardized to two standard deviations) to control for the effect of sample collection time on our response variable. Reproductive phases (pregnancy, lactation) and parity (primiparous, multiparous) were included as parametric predictor terms to account for the average effect of each level of the categorical predictors on the response variable, assuming a linear relationship (testing variance inflation factors using the ‘vif’ function of the ‘car’ package (Fox & Weisberg, 2019) showed no issues with multicollinearity). Furthermore, we included smooth terms for the response variable across the days relative to birth (mean-centred and standardized). We specified these terms so that the model calculated different smooths across the days relative to birth for each reproductive phase and for each parity level. In other words, the model fit separate smooths across days during pregnancy (−245 to 0) and across days during lactation (1 to 667) and for primiparous and multiparous females. We performed a grid search (between 5 and 50) to find the best smoothing parameter values that minimize prediction error, thus optimizing the model’s performance while avoiding overfitting. Furthermore, we included a random smooth term for female identity to account for the repeated sampling nature of our data set and to allow for individual variation in the trajectories of the curves. As only one female was included in the data set with two parity states (primiparous and multiparous) and we expected different intercepts depending on parity, we included a separate intercept for each parity state for this female.

Model assumptions and model settings were checked using the ‘gam.check’ (package ‘mgcv’) and ‘acf_resid’ functions (detection of autocorrelation, package itsadug; van Rij et al., 2022). To compare models, we used the ‘compareML’ function (package ‘itsadug’). For plotting the smooths of log-transformed CTX-I across the days relative to the date of birth by reproductive phase (pregnant, lactating) and parity (primiparous, multiparous), we used the ‘plot_smooth’ function of the package ‘itsadug’.

To investigate differences in urinary CTX-I levels between the categorical reproductive phases variable (phases: before pregnancy, first trimester of pregnancy, second trimester, third trimester, and early and late lactation), we fitted a linear mixed model with a Gaussian error structure (Grafen & Hails, 2002). Log-transformed urinary CTX-I levels were the response variable. Time of sample collection (mean-centred and standardized to two standard deviations in the new data set) was entered as a control variable. We added a random intercept for the individual to control for repeated measures of females and to estimate the variance explained by interindividual differences. Furthermore, we included a predictor variable, ‘condition’, indicating whether a measurement was sampled from before pregnancy, from the first, second, or third trimester during pregnancy, or from after pregnancy (Table S2). Likelihood ratio tests were used to compare the full model to the null model (including only the random effect and the control variable time of sample collection; χ^2^ = 40.20, df = 5, *p* < 0.001). We visually inspected quantile–quantile plots and the distribution of residuals plotted against fitted values and found that model assumptions were met. Models were run using the ‘lme4’ package v1.1-34;15 (Bates et al., 2015). Post-hoc tests comparing different reproductive phases of the variable condition were performed using the ‘emmeans’ package (Lenth, 2021).

## 3. Results

### 3.1. Urinary CTX-I levels increased during pregnancy in zoo bonobos – biological validation

**Figure 2.**
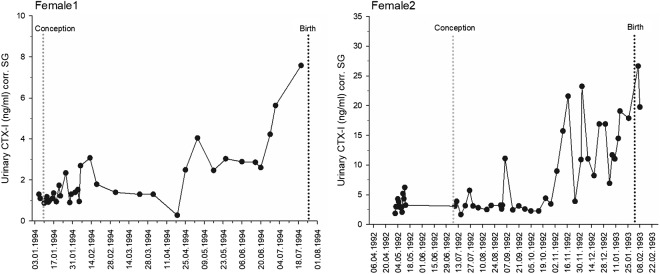
Urinary CTX-I levels in two zoo-housed female bonobos during pregnancy. SG = specific gravity. *y*-Axes display different scales to better visualize data within individuals. The light grey dashed line at −245 indicates the estimated day of conception based on the average gestation length in bonobos (Stevens, 2020). The black dashed line indicates the day of birth.

In the zoo-housed bonobos, urinary CTX-I levels increased during pregnancy, approximately 3 months before parturition (Figure 2). In one female, levels increased by a factor of 4 during this period, whereas in the other female, levels increased by a factor of 10 (Figure 2, note *y*-axis scaling).

### 3.2. Urinary CTX-I levels during pregnancy and lactation in wild bonobos

The grid search revealed that specifying the smooth terms across days relative to offspring birth with eight basis functions minimized the cross-validation error while avoiding overfitting. Consequently, we ran our main model with the predictor variables reproductive phase (pregnant, lactation), parity (primiparous, multiparous), and smooths over days relative to birth for reproductive phase and parity (Table 1). This model differed significantly from the corresponding null model (χ^2^ = 6.50, df = 2, *p* = 0.002) and provided a better fit (AIC difference − 40). Model assumptions were met and autocorrelation of residuals was negligible.

**Table 1.** Results of the generalized additive mixed model on CTX-I levels across pregnancy and lactation in primiparous and multiparous female bonobos. For the parametric predictor variables, estimates (β), standard errors (SE), *t*-values and *p*-values are presented. For the smooth terms, effective degrees of freedom (edf), reference degrees of freedom (ref.Df) and *F*-statistic are provided

Factor variables	Reference category	β	SE	*t*-Value	*p*-Value
Intercept		1.820	0.108	16.821	
Reproductive phase	Lactation	−1.294	0.384	−3.369	0.001
Parity	Primiparous	0.434	0.143	3.024	0.003
Sampling time		−0.710	0.100	−7.112	<0.001
**Smooth term variables**	**Reference category**	**Edf**	**Ref.df**	***F*-value**	***p*-Value**
*Days relative to birth*					
Reproductive phase	Lactation	2.798	3.176	4.226	0.003
Parity	Primiparous	3.415	4.126	1.993	0.092
*Random smooth*					
Female ID		4.416	9.000	1.120	0.019
**Adjusted *R*^2^ (deviance explained)**		0.294 (32.6%)			
***N* (*p*-value)**		296 (0.002)			

**Figure 3.**
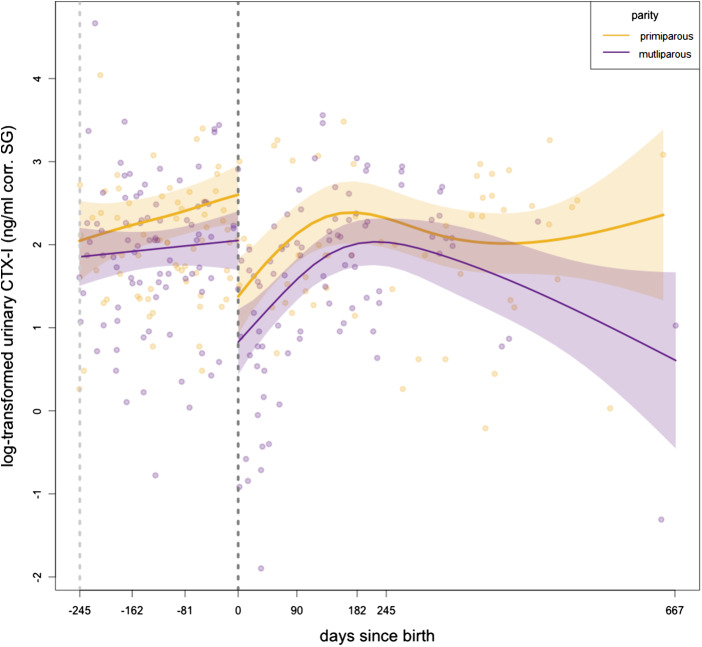
Smooths of log-transformed urinary CTX-I levels across days relative to offspring birth for pregnant (days −245 to 0) and lactating wild female bonobos (days 0 to 667). trajectories for primiparous (gold) and multiparous (purple) females are shown. Urinary CTX-I levels were corrected for specific gravity (SG) and then log-transformed. On the *x*-axis, the days relative to offspring birth are displayed. The light grey dashed line at −245 indicates the estimated day of conception based on the average gestation length in bonobos. The black dashed line at 0 indicates the day of birth.

Overall, the model revealed that urinary CTX-I levels were higher in pregnant than in lactating females and that primiparae had higher levels than multiparae (Table 1, Figure 3). The smooth terms differed between reproductive phases. Throughout pregnancy, urinary CTX-I levels remained relatively constant, particularly in multiparous females. Primiparous females showed a slight increase in urinary CTX-I levels towards the end of the pregnancy, with the highest levels before giving birth. With the onset of lactation, females showed a marked decrease in urinary CTX-I levels that then increased within the first 6 months of lactation.

**Figure 4.**
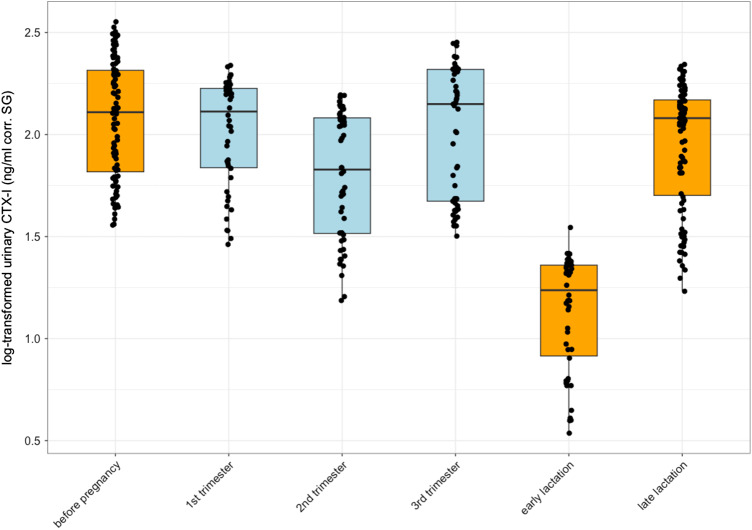
Differences in log-transformed urinary CTX-I levels by reproductive state in wild bonobos: before pregnancy (female is cycling, in orange), first trimester, second trimester, third trimester (pregnant in blue), early lactation (up to 90 days after offspring’s birth, in orange), and late lactation (>91 days after offspring’s birth, in orange). The central solid black line within each box represents the median. The top and bottom edges of the box represent the upper (75th percentile) and lower quartiles (25th percentile), respectively. The whiskers from the box indicate the range of data within 1.5 times the interquartile range from the quartiles. Individual data points are overlaid with a jitter effect, making them easier to distinguish and providing additional detail about the distribution of data points across conditions.

As the decrease in urinary CTX-I levels at the beginning of the lactation period was unexpected (for comparison with other mammalian patterns, see Figure 1), we performed further analyses to understand whether urinary CTX-I levels returned to a baseline level. These analyses revealed that urinary CTX-I levels were lower in the early lactation state than in any other reproductive phases (Table 2, Figure 4, and Table S3 show means, standard deviations (SD), medians and ranges (lowest to highest value) of log-transformed CTX-I (ng/ml corr. SG) levels by reproductive state).

**Table 2.** Results of the post-hoc pairwise comparisons of urinary CTX-I levels during reproductive state (before pregnancy, first, seconf, and third trimester of pregnancy, early and late lactation) in female bonobos

Contrast	β (estimate)	SE	Df	*t*-Ratio	*p*-Value
First to second	0.097	0.174	388	0.558	0.994
First to third	−1.148	0.173	384	−0.857	0.956
First – before	−1.136	0.157	389	−0.870	0.954
First – early	0.823	0.179	390	4.590	<0.001
First – late	0.052	0.154	390	0.339	0.999
Second to third	−0.245	0.168	388	−1.466	0.686
Second – before	−0.233	0.151	390	−1.552	0.631
Second – early	0.725	0.176	391	4.130	<0.001
Second – late	−0.045	0.149	391	−0.304	0.9997
Third – before	0.012	0.149	386	0.082	1.000
Third – early	0.971	0.175	391	5.540	<0.001
Third – late	0.200	0.147	388	1.363	0.749
Before – early	0.959	0.159	384	6.017	<0.001
Before – late	0.188	0.126	390	1.492	0.670
Early – late	−0.771	0.156	386	−4.944	<0.001

## 4. Discussion

Our study represents the first measurement of a bone resorption marker during pregnancy and lactation in a wild mammal living under natural conditions, as well as in a great ape species with prolonged maternal care. By focusing on wild bonobos, we can consider these findings within a natural and evolutionarily relevant context that can be compared to previously published patterns in humans, captive primates, and domesticated mammals (Kovacs, 2011; Liesegang et al., 2006; Van Riet et al., 2016). We predicted that urinary CTX-I levels, a marker of bone resorption, would increase during pregnancy and peak at the end, reflecting an increase in bone turnover following patterns found in, albeit limited, studies of humans and captive/domesticated mammals. This hypothesis was supported. Our investigation revealed an expected increase towards the end of pregnancy in zoo-housed and primiparous wild females. These findings biologically validate our measurements and further support the notion that pregnancy influences bone metabolism, likely as part of the broader physiological adaptations to meet foetal skeletal growth demands (Kovacs, 2011).

In human studies, higher urinary CTX-I levels were found during lactation compared with pregnancy or cycling levels, based on the assumption that lactation involves increased maternal bone resorption to meet calcium demands for milk production (Kovacs, 2011; Yamaga et al., 1997). In wild bonobos, urinary CTX-I levels did not further increase during lactation, as found in other domesticated or captive mammals (Liesegang et al., 2007; Yamaga et al., 1997). Instead, CTX-I levels decreased, with levels in early lactation (the first 3 months) significantly lower than those during pregnancy and all other reproductive phases. This unexpected divergence challenges the assumption that lactation increases bone resorption to meet calcium demands and raises questions about the potential physiological or behavioural mechanisms that bonobos use to counteract bone loss and the utility of current comparative data from human and domesticated animal populations. We discuss each of these findings in more detail below.

We found increased urinary CTX-I levels at the end of pregnancy in zoo-housed bonobos and wild-living primiparous bonobos. This result aligns with human studies, where bone turnover markers, including serum CTX-I levels, are elevated in the third trimester (Cross et al., 1995; Gallacher et al., 1994; Kaur et al., 2003), and with findings in domesticated mammals, such as cows, where increased bone resorption occurs around parturition (Liesegang et al., 2000). In the two zoo bonobos studied, urinary CTX-I levels increased 4- to 10-fold during pregnancy, indicating a pronounced rise in bone resorption. Comparatively, studies in humans report a two- to threefold increase in urinary CTX-I levels during pregnancy (Curtis et al., 2021; Hannon & Eastell, 2000; Yamaga et al., 1997). Although the magnitude of the increase in bonobos is higher than the average reported for humans, it still falls within the broader range of variation observed across individuals and studies, for example domesticated animal studies (Liesegang et al., 2006, 2007). These findings suggest that bonobos exhibit physiological responses to pregnancy that are comparable to those seen in humans, albeit with some species-specific variability. Additionally, differences in normalization methods – such as the use of specific gravity (SG) in our study versus creatinine normalization in human studies – may contribute to differences in the observed fold increase in urinary CTX-I levels.

By contrast, urinary CTX-I levels in wild multiparous female bonobos remained constant throughout pregnancy. It is possible that the effect of bone resorption is only large enough to be detected in primiparous, often younger, females, as human bone mass density losses at the end of pregnancy are greater in adolescents than in adult women (Bowman & Miller, 2001). Zoo-housed bonobos experience an adolescent growth spurt, and afterwards bone growth slows around 13–15 years of age (Behringer et al., 2024, 2016; Berghaenel et al., 2023). Although the exact ages of the primiparous wild females in our study remain unknown due to female dispersal, it is possible that some females were still growing, which could have influenced their bone resorption patterns during reproduction. This could also explain the comparable pattern in the two zoo-housed bonobos, as both were multiparous but under 16 years old when the samples were collected. If changes in CTX-I levels occur during pregnancy in older, multiparous females, they may be minor compared with primiparae and could fall within the large daily intra-individual variation (Behringer et al., 2024). Previous non-human primate studies found that pregnancy reduced bone mass in primiparous pig-tailed macaques (Ott et al., 1999), in contrast to female long-tailed macaques in a breeding colony (Lees et al., 1998). Both studies speculated that the maternal skeleton might not be the primary source of calcium for the developing foetus and that calcium demands during pregnancy are primarily met by increased intestinal calcium absorption (Lees et al., 1998; Ott et al., 1999). In our study population, it is conceivable that multiparous females also cover the calcium needs of foetal growth through intestinal and renal calcium absorption. The assumption that calcium absorption may be pivotal in primate pregnancy is indirectly supported by research demonstrating increased feeding time (*Papio anubis, Presbytis entellus, Pan troglodytes schweinfurthii*) (Barton, 1990; Koenig et al., 1997; Murray et al., 2009) and/or improved food quality during pregnancy in natural conditions across several primate species (*P. troglodytes schweinfurthii, Saimiri oerstedi, Varecia rubra*) (Boinski, 1988; Murray et al., 2009; Vasey, 2005). These nutritional changes, driven by increased energy needs, may allow pregnant females to cover specific nutritional demands of pregnancy that, in turn, limit calcium resorption from the skeleton. Such adaptive behavioural changes align with dietary patterns observed in human populations, such as the Aché and nomadic !Kung, who exhibit shifts in foraging strategies and food intake in response to reproductive and energetic demands (Hill & Hurtado, 1996; Kolata, 1974). Therefore, human studies and our results suggest that pregnant females in natural conditions may be able to meet increasing calcium demands through adaptive changes to feeding duration or food quality, thereby increasing intestinal calcium availability and preventing bone loss and the associated increase of CTX-I levels.

In most human studies cited, it is not explicitly reported whether participants used supplements or avoided them (Augustine et al., 2023; Carneiro et al., 2010; Gallacher et al., 1994; Yamaga et al., 1997). However, the consistent observation of increased CTX-I levels during pregnancy in modern human populations suggests that either (1) many women may not receive or use supplementation or (2) that these increases occur even with supplementation. For example, studies investigating the effects of supplementation, such as vitamin D (Curtis et al., 2021; Krupa et al., 2024), have shown that supplementation moderates, but does not entirely prevent, the rise in CTX-I levels during pregnancy in humans. This finding may imply that single-nutrient supplementation is insufficient, as multiple nutritional factors relevant to bone homeostasis likely interact during pregnancy. Moreover, studies on calcium supplementation during pregnancy have yielded contradictory results. For example, one study reported that 1200 mg/day of calcium carbonate significantly reduced bone resorption in pregnant Mexican women and at one month postpartum (Ettinger et al., 2014), whereas another study observed the opposite effect in Gambian women (Jarjou et al., 2010). These discrepancies likely reflect differences in baseline nutritional status, dietary composition, and other factors affecting calcium absorption and bone turnover. Finally, the highly processed nature of modern diets in the global north, even when supplemented, may fail to meet the complex and dynamic nutritional demands of pregnancy. This limitation could explain why increased bone turnover is still observed during pregnancy despite access to supplementation in humans today.

By contrast to patterns found in previous studies of humans and other mammals, wild bonobo urinary CTX-I levels decreased, rather than increased, at the onset of lactation. Although wild bonobos showed an increase in CTX-I levels after 3 months postpartum, these levels never exceeded those measured during pregnancy. Urinary CTX-I levels during lactation were comparable between primiparae and multiparae, although primiparae exhibited higher overall CTX-I levels. Similarly, in goats and sheep, bone loss during second pregnancies and lactation was less pronounced than during the first reproductive cycle (Liesegang et al., 2007). In line with our results, lower bone resorption markers have been observed in multiparous goats, sheep, and monkeys (Liesegang et al., 2007; Ott et al., 1999).

The surprising decrease in urinary CTX-I levels during early lactation contradicts our expectation of substantial bone loss to meet the calcium requirements of a growing infant, as has been described in other mammals, including primates (Miller et al., 1989; Lees et al., 1998; Liesegang et al., 2007; Tojo et al., 1998; Vajda et al., 1999; Van Riet et al., 2016; Watts et al., 2021). Although urinary CTX-I levels in one human study declined throughout the course of lactation, the levels were nearly as high immediately after birth and three months later as they were during pregnancy (Kaji et al., 2007; Figure 1). In our study, the female bonobos notably did not show high CTX-I levels in the first months following birth. This may suggest a potential inhibition of osteoclast activity. Several physiological and behavioural mechanisms might explain the decrease in bone resorption in wild bonobos during early lactation. One physiological hypothesis to explain this result is that bonobos conserve energy by reducing bone resorption during this energy-demanding reproductive phase. That lactation is such an energy demanding period is indicated by increased cortisol levels in bonobos (Nurmi et al., 2023) and other primate species (*Cercopithecus mitis, Macaca assamensis*) (Foerster et al., 2012; Touitou et al., 2021), as cortisol is a key hormone involved in energy metabolism. Cortisol mobilizes glucose and other energy reserves to meet immediate physiological needs (Cooper et al., 2016). Bone remodelling is an energy-demanding process, and given that the skeleton is one of the largest organs in the mammalian body, it likely incurs high costs (Ducy, 2011). Reducing bone remodelling could be a strategy to conserve energy during the energy-intensive lactation period. The mechanism behind such a reduction in bone remodelling could be genetic: the downregulation of genes associated with bone resorption in hibernating bears has been shown to preserve bone density during long periods of inactivity (Goropashnaya et al., 2021), demonstrating that gene regulation can prevent bone loss during specific periods of the life cycle. Although this strategy may potentially affect calcium availability for breastmilk production if compensatory mechanisms are insufficient, the primary focus here is to explore the possibility that osteoclast activity may be genetically regulated as an adaptive energy-conservation strategy during lactation. Future studies of the mineral content of breastmilk in captive non-human primates may provide additional information to test this hypothesis (Hinde & Milligan, 2011; Milligan, 2007). For example, whereas zoo-housed *P. troglodytes* produce milk with lower calcium content (0.021 g/100 ml) compared to humans (0.033 g/100 ml), *Gorilla gorilla* have similar levels (0.034 g/100 ml) (Milligan, 2007). However, no comparable data exist for bonobos, either in zoos or in the wild, and given ethical challenges, such data are unlikely to be collected in the future in any wild ape species.

Previous studies have shown that females can regain the bone mass lost during pregnancy and lactation, but this replenishment of skeletal reserves is possibly species-specific and can be affected by differences in the length of the lactation periods (Van Riet et al., 2016). In human studies, where the average bone loss is 1–3% per lactation month (Kovacs, 2005), pre-pregnancy levels of bone density typically recover within 6–12 months after weaning (Kovacs, 2011). In monkeys (*M. fascicularis, C. aethiops*) with lactation periods ranging from one to several months, recovery from lactation-induced bone loss takes up to 10 months postpartum or longer (Hiyaoka et al., 1996; Lees et al., 1998). Furthermore, in other primates (*M. mulatta, Leontocebus fuscus*), females struggle to recover bone minerals before subsequent pregnancies (Cerroni et al., 2003; Power, 1991). Our results suggest that bonobos may have a strategy for replenishing skeletal reserves that differs from what is known in other primates. Wild bonobos nurse their offspring for up to 4–5 years (Oelze et al., 2024) and have an average interbirth interval of around 5.4 years (Tkaczynski et al., 2020). Perhaps female bonobos, and possibly other wild primates, aim to minimize bone resorption at the beginning of this calcium-demanding period to ensure bone density is not depleted in order to reduce fracture-risk levels over longer lactation periods.

The decrease in bone resorption during early lactation in wild bonobos may also result from feeding adaptations. Increased calcium intake during lactation has been shown to improve maternal bone health by replacing maternal bone mass and reducing bone resorption in humans (reviewed in Thomas & Weisman, 2006). Therefore, as suggested above in relation to stable CTX-I levels during late-stage pregnancy in multiparae, adapting feeding behaviour and dietary quality during lactation might explain the unique CTX-I patterns observed in wild bonobos. Field observations of several primate species indicate that lactating females increase their intake of high-energy foods, overall food energy, and time allocated to foraging, particularly when food quality is poor (e.g. *Saimiri sciureus, Theropithecus gelada, Cebus capucinus, Callicebus cupreus*) (Boinski, 1988; Dunbar et al., 2002; McCabe & Fedigan, 2007; Tirado Herrera & Heymann, 2004). Although there are currently no published data on food quality or quantity changes in wild bonobos during lactation, food items often consumed by male and female bonobos are high in calcium (see table 1 in Hohmann et al., 2019). If bonobos generally have a high-calcium diet, or if females increase their calcium intake during lactation, this can have physiological effects that limit bone resorption. Increased calcium absorption can lead to increased calcitonin release, which inhibits osteoclast activity (Taylor et al., 1975; Woodrow et al., 2006). Additionally, high blood calcium levels prevent the secretion of parathyroid hormone, which stimulates osteoclast activity (Rejnmark & Ejlsmark-Svensson, 2020). Both mechanisms would protect the maternal skeleton from excessive resorption during lactation. Other dietary adjustments, such as consuming antioxidant-rich foods containing vitamin E and polyphenols, have been shown to help combat oxidative stress and reduce bone resorption markers like CTX-I (Mottaghi & Nasri, 2021). Further investigation is needed to determine whether any of these mechanisms are involved in wild bonobos during lactation. Importantly, however, zoo-housed females (or domesticated/captive mammals in general) lack the autonomy to display such behavioural adaptations due to environmental constraints or the absence of key ecological cues that might otherwise induce physiological or epigenetic plasticity. This raises the possibility that the inability to respond to changing metabolic demands through natural behavioural adjustments, rather than diet or activity levels alone, contributes to the observed differences in bone turnover between wild and captive individuals.

We speculate that the urinary CTX-I pattern in wild female bonobos reflects the unique dietary and environmental conditions of their habitat. These conditions differ from those of human populations in industrialized societies, as well as from those of domestic and laboratory animals, particularly in terms of food availability, activity patterns, and the duration of lactation. Our results highlight the value and importance of understanding the reproductive ecology of wild non-human primates living in their natural environments, particularly with regard to the costs of pregnancy and lactation on maternal bone loss.

This sentiment also applies to non-industrialized human groups, for which there is substantial variability in the duration of exclusive breastfeeding (Dupras et al., 2001; Richards et al., 2002; Sellen, 2007) and diverse breastfeeding practices (Fink et al., 1992; Gray, 1994; Kolata, 1974), yet nothing is known about the potential effects on bone density. For example, previous studies directly measuring bone structure from skeletal remains have shown that trabecular bone density (Chirchir et al., 2015, 2017; Ryan & Shaw, 2015), cortical bone thickness, and cortical strength (Ruff & Larsen, 2014; Shaw & Stock, 2013; Stock & Pfeiffer, 2004) are higher in more active or mobile foraging populations compared to agriculturalists or recent humans. However, it is unclear if this increased skeletal robusticity is found in contemporary hunter-gatherer or pastoralist human groups with more active lifestyles and natural diets and, if so, if they experience the same loss in bone mass during reproductive phases as documented in global north populations. For instance, human foragers tend to avoid noncommunicable diseases and promote healthy ageing through high activity levels, but these benefits are counterbalanced by increased wear-and-tear, risk of injury, and energy constraints, especially during reproduction when energy is diverted toward pathogen defence and reproduction (Stieglitz, 2024).

Our results add to this complexity by demonstrating that the bone density costs associated with pregnancy and lactation, previously documented in industrialized humans and domesticated/captive mammals, are not representative of wild-living bonobos and perhaps other primates living in natural environments. Understanding these physiological mechanisms in wild bonobos could offer profound insights into natural strategies for bone preservation. Such knowledge could inform approaches to reduce osteoporosis in captive animals (Lennox & Goodship, 2008) and women from industrialized societies, where modern lifestyles and reproductive patterns often disrupt traditional bone metabolism processes. By learning from the adaptive strategies of wild bonobos and other hominoids, we may identify more effective ways to mitigate bone density loss associated with reproduction and ageing in human populations.

## 5. Conclusions

Our study provides the first results of bone metabolic changes of wild bonobos during pregnancy and lactation, indicated by urinary CTX-I levels. We observed unique patterns in wild bonobos, with the lowest CTX-I levels during lactation. These levels are in contrast with those reported in human populations and captive mammals, highlighting the distinct adaptive strategies employed in natural environments. The decrease in urinary CTX-I levels during early lactation challenges existing assumptions about bone resorption dynamics in primates, suggesting potential behavioural and physiological adaptations to conserve bone mass under calcium-demanding conditions. These findings not only highlight unique adaptive strategies to conserve skeletal integrity under calcium-demanding conditions, but also offer evolutionary context for understanding the trade-offs between reproduction and skeletal health in primates, including humans and early hominins.

Our findings underscore the importance of studying non-human primates in their natural habitats to understand the full spectrum of reproductive costs on skeletal health. Further investigations into the dietary and behavioural mechanisms influencing bone health in wild bonobos could pave the way for innovative approaches to mitigate bone density loss associated with reproduction and ageing in humans.

## Supporting information

Behringer et al. supplementary material Behringer et al. supplementary material

## Data Availability

Data are available at GRO.data, where the source data and R code are permanently stored.

## Supplementary material

The supplementary material for this article can be found at https://doi.org/10.1017/ehs.2025.10013.

## Research transparency and reproducibility

Source data for statistics and figures as well as used R code in the paper is permanently stored at GRO (“Replication Data for: Wild bonobos experience unusually low bone resorption during early lactation relative to humans and other mammals”, https://doi.org/10.25625/QSKFW4).
