# Supplementary material for: Wild bonobos experience unusually low bone resorption during early lactation relative to humans and other mammals
Source: Evol Hum Sci. 2025 Jul 22;7:e27. doi: 10.1017/ehs.2025.10013 (PMC12516605; doi:10.1017/ehs.2025.10013)
Supplement: Behringer et al. supplementary material [file S2513843X25100133sup001.docx]

**Supplementary Material:**

Wild bonobos experience unusually low bone resorption during early lactation relative to humans and other mammals

**Supplementary Methods**

*CTX assay description*

All samples were first diluted 1:5 using CrossLaps Standard 0 and then run in duplicates on the assay plate following instructions from the IDS company, East Boldon, United Kingdom. In brief, 10 µl of standards, controls, and (diluted) samples were pipetted into streptavidin-coated microtiter wells. Subsequently, 150 µl of an antibody solution (a mixture of biotinylated antibody and peroxidase-conjugated antibody) was added to each well, and the plate, sealed with tape, was incubated for 120 minutes at room temperature, in the dark, on a shaker at 300 rpm. After incubation, the wells were emptied and washed five times with 300 µl of washing buffer. Then, 100 µl of a chromogenic substrate was added to each well, sealed, and incubated at room temperature, in the dark, on a shaker for 15 minutes. Finally, 100 µl of stop solution (sulfuric acid) was added to each well to stop the color reaction. Absorbance was measured at 450 nm with 650 nm as a reference on a plate reader.

**Table S1**

Number of samples for each female and pregnancy per reproductive phase. Primi. = primiparous; multi. = multiparous

| **Female** | **parity** | **Collection period** | **Before pregnancy** | **1st trimester** | **2nd trimester** | **3rd trimester** | **Early lactation** | **Late lactation** | **Total** |
| --- | --- | --- | --- | --- | --- | --- | --- | --- | --- |
| Bella | primi. | May 2019–Sept. 2022 | 11 | 4 | 8 | 6 | 2 | 3 | **34** |
| Djulie | primi. | Jan. 2014–Feb. 2022 | 9 | 2 | 5 | 3 | 1 | 2 | **22** |
| Gwen | multi. | Jan. 2011–Dec. 2013 | 6 | 5 | 4 | 3 | 1 | 8 | **27** |
| Lombe | primi. | March 2018–Nov. 2021 | 9 | 2 | 1 | 2 | 3 | 12 | **29** |
| Ngola | primi. | Oct. 2018–Nov. 2021 | 5 | 5 | 5 | 6 | 3 | 9 | **33** |
| Olga | multi. | Dec. 2011–Dec. 2013 | 7 | 5 | 3 | 4 | 11 | 6 | **36** |
| Paula | multi. | Dec. 2011–April 2013 | 3 | 6 | 5 | 4 | 11 | 8 | **37** |
| Polly | primi. | Jan. 2012–Nov. 2015 | 13 | 5 | 2 | 10 | 3 | 18 | **51** |
| Polly | multi. | Feb. 2016–Nov. 2019 | 7 | 1 | 2 | 3 |  | 4 | **17** |
| Rio | multi. | April 2011–April 2013 | 10 | 3 | 11 | 5 | 5 | 19 | **53** |
| Uma | multi. | Sept. 2011–April 2013 | 8 | 8 | 6 | 7 | 6 | 10 | **45** |
| **Total** |  |  | **88** | **46** | **52** | **53** | **46** | **99** | **384** |

**Table S2**

Results of the linear mixed model. The model contained the main effects of the categorical condition variable (before pregnancy, 1^st^ trimester, 2^nd^ trimester, 3^rd^ trimester, early lactation, late lactation), the time of sample collection (z-transformed minutes since midnight), and a random intercept for the individual. Estimates (β) and standard errors (SE) of individual variables are presented. For the categorical variable condition, the reference category is indicated.

|  | Reference category | β (estimate) | SE |
| --- | --- | --- | --- |
| Intercept |  | 2.089 | 0.097 |
| Time (z-transformed) |  | -0.611 | 0.090 |
| Condition: 1^st^ trimester | Before pregnancy | -0.136 | 0.155 |
| Condition: 2^nd^ trimester | Before pregnancy | -0.234 | 0.149 |
| Condition: 3^rd^ trimester | Before pregnancy | 0.012 | 0.147 |
| Condition: early lactation | Before pregnancy | -0.959 | 0.156 |
| Condition: late lactation | Before pregnancy | -0.188 | 0.125 |
|  |  |  |  |
|  | Variance | Standard deviation |  |
| Individual female | 0.012 | 0.110 |  |
| Residual | 0.712 | 0.843 |  |

**Table S3**

Descriptive statistics of log-transformed CTX-I (ng/ml corr. SG) levels by reproductive state (condition). CTX-I = C-terminal crosslinking telopeptide of type I collagen; SG = specific gravity; SD = standard deviation.

| **Condition** | **Mean** | **SD** | **Median** | **Range** |
| --- | --- | --- | --- | --- |
| Before pregnancy | 12.57 | 15.17 | 7.76 | 0.54–98.58 |
| 1st trimester | 12.19 | 17.20 | 8.04 | 1.11–106.17 |
| 2nd trimester | 7.51 | 4.63 | 6.63 | 0.46–21.67 |
| 3rd trimester | 10.00 | 7.69 | 8.12 | 1.04–31.20 |
| Early lactation (up to 3 months) | 5.38 | 6.04 | 3.57 | 0.15–26.00 |
| Late lactation | 9.53 | 7.14 | 7.80 | 0.27–35.10 |

**Figure S1**. Density plot of urine samples collected from 10 females during 11 periods of lactation. Urine samples of mothers were collected from the first day after birth onwards. The majority of samples from lactating females were collected within the first six months.
